# Reliability and diagnostic performance of a new blood ketone and glucose meter in humans

**DOI:** 10.1186/s12970-020-00404-2

**Published:** 2021-01-07

**Authors:** Andrew Ray Moore, Angelia Maleah Holland-Winkler, Jenna Kate Ansley, Eric Deiondre Hunter Boone, Megahn Kimberanne O’Reilly Schulte

**Affiliations:** Department of Kinesiology, Augusta University, 3109 Wrightsboro Road, Augusta, GA 30909 USA

**Keywords:** Beta-hydroxybutyrate, Blood meter, Keto-mojo, Precision Xtra, Substrate

## Abstract

**Background:**

Accurate and reliable monitoring of blood ketone and glucose levels is useful for athletes adhering to a ketogenic diet who want to verify that they are in a state of ketosis and, therefore, accruing performance adaptations. However, the cost of devices and testing materials may prohibit their use. More affordable field testing systems are available, but their accuracy and reliability remain in question. The objectives of this study were to evaluate the agreement between a previously validated ketone and glucose meter (Meter 1 – Precision Xtra) and a more affordable meter that has not been validated (Meter 2 – Keto-Mojo), and also to assess the diagnostic performance of Meter 2 for identifying nutritional ketosis.

**Methods:**

Thirteen participants (7 females and 6 males; 21.6 ± 3.0 years old) visited the laboratory three times in this randomized, double-blind cross-over design study. Ketone and glucose levels were measured with Meter 1 and Meter 2 twice before and twice after ingestion of a racemic ketone, natural ketone, or maltodextrin supplement. Intraclass correlation coefficient (ICC) estimates and their 95% confidence intervals were calculated to evaluate interrater reliability for Meter 1 and Meter 2. Bland-Altman plots were constructed to visually assess the agreement between devices. Area under the ROC curve analysis was performed to evaluate the diagnostic ability of Meter 2 to detect nutritional ketosis at a threshold ketone level of 0.5 mM as identified by Meter 1.

**Results:**

Reliability between the meters was excellent for measuring ketones (ICC = .968; .942–.981) and good for measuring glucose (ICC = .809; .642–.893), though the Bland-Altman plot revealed substantial differences in agreement for measuring glucose. Area under the ROC curve (Area = 0.913; 0.828–0.998) was excellent for diagnosing nutritional ketosis.

**Conclusions:**

Both Meter 1 and Meter 2 displayed excellent agreement between each other for ketone measurement. Meter 2 also displayed an excellent level of accuracy for diagnosing nutritional ketosis at a threshold value of 0.5 mM, making it an effective and affordable alternative to more expensive testing devices.

## Introduction

The ketogenic diet (KD) is a nutritional approach in which daily carbohydrate intake is restricted, commonly to < 50 g/day or 5% of energy intake [1–3]. Chronic daily restriction of carbohydrates limits the use of glucose as a substrate and necessitates the use of free fatty acids (FFAs) for energy. FFAs cannot be directly used as substrate in the absence of oxaloacetate (a byproduct of glycolysis), and must instead be converted to ketone bodies in the liver through a process called ketogenesis [4]. FFAs are converted to acetyl-CoA and then to ketone bodies (KB), most notably in the form of β-hydroxybutyrate (BHB), which can then be used by tissues of the body as an alternative fuel source to glucose [5]. Prolonged adherence to the KD forces the body to adapt to these changes in substrate availability and shift to a state of ketosis in which most energy is derived from ketones (via FFAs) rather than from glycogen [4]. Adoption of the KD to induce a state of ketosis has been historically associated with the treatment of pathological conditions such as epilepsy and diabetes [1]. However, there is recent interest in applying this diet to various athletic populations to improve sport performance as well [for reviews, see [6–8]].

The metabolic changes that characterize the KD reduce the dependence of athlete performance on limited carbohydrate stores and promote glycogen sparing during prolonged activity, a possible ergogenic benefit [2, 9]. This benefit is most relevant to KD adapted athletes competing for substantial periods of time (> 2 h) such as ultra-endurance runners in whom fat oxidation contributes a greater percentage of energy, but who retain the ability to use muscle glycogen as fuel when needed [10]. Reduced tissue inflammation has been observed as well, which complements recovery from practice for subsequent athletic competition [11–13]. In addition to observed metabolic and recovery adaptations, adherence to the KD causes changes in body mass and composition which may be favorable to competition in many weight-class sports. Among these are an initial rapid reduction in body weight (due to decreased glycogen and water storage throughout the body and appetite reduction from hormone actions) and increased fat oxidation [6, 14].

The characteristic metabolic and body composition changes following KD adherence are practical and effective for a variety of athletes. Those who compete in specific weight categories (wrestling/martial arts, Olympic weightlifting, etc.) benefit from being able to shed weight quickly to meet these category requirements without inducing common weight-cutting side effects, such as dehydration or loss of skeletal muscle protein content [15]. Strength and power athletes may also benefit by dropping unnecessary weight and maintaining muscular strength and power, thereby increasing strength-to-mass ratio, provided that protein consumption is sufficient [6, 14]. Even endurance athletes may benefit from adhering to a ketogenic diet, through improvements in peak power, VO_2_max, lactate threshold, and markers of muscle damage [10, 13, 16].

Accurately monitoring BHB levels is important for maintaining diet adherence and making effective modifications to the diet. Desired metabolic adaptations to a ketogenic diet are made when the athlete is in a state of nutritional ketosis (NK) and BHB is increased above resting levels to at least 0.5 mM in the blood [17]. Objective measurement of BHB levels is needed to confirm a state of NK, or to make necessary changes to the diet and activity level to achieve NK and resulting adaptations throughout the training cycle. Knowledge of blood glucose levels is also of interest when tracking metabolic adaptations, as glucose levels remain at normal physiological levels following ketogenic adaptation and are still used as substrate during exercise [18].

The gold standard of ketone measurement is mass spectrometry, a method that is both expensive and inaccessible to most athletes. One of the most commonly used high-performing field measurement device is Abbott Labs’ Precision Xtra glucose/ketone monitor system (Meter 1). This device has been validated against the gold standard laboratory method in animals [19, 20] and previous models of the device have been found acceptable for human use at ketone levels below 3 mM [21, 22], a commonly reported blood ketone level in persons adhering to the KD [23]. With daily utilization, this method may become expensive due to the cost of the disposable test strip needed with each measurement. Conversely, urinary ketone tests are freely available and quite cheap, yet are often reported to be unreliable [24]. Urinary analysis can only be used to measure acetoacetate, a more unstable KB than BHB. KBs are also detected in the blood before they are detected in the urine (which can vary in terms of concentration and volume), making urinary analysis of ketones unreliable and limit their accuracy [24]. Thus, there is a need for more valid and reliable blood ketone and glucose monitoring systems that are also affordable and accessible to athletes.

Keto-Mojo (Meter 2) is a relatively new measurement system that is considerably more affordable to use than Meter 1. For manufacturer-sold products, the cost of Meter 2 test strips for ketone and glucose testing are 1/5 and 1/6 the price of test strips for Meter 1, respectively. However, the reliability and practical value of Meter 2 must be assessed prior to use in healthy human populations. Therefore, the objectives of this study were: (i) to evaluate the more affordable Meter 2 and compare it with Meter 1 to assess reliability and agreement for BHB and glucose measurement in healthy human subjects; and (ii) to assess the diagnostic performance of Meter 2 vs. Meter 1 for identifying NK at a level of BHB = 0.5 mM. We hypothesized that Meter 1 and Meter 2 would yield similar glucose and ketone readings for healthy individuals and diagnostic performance of Meter 2 would be acceptable for identifying NK compared to Meter 1.

## Methods

### Experimental design

A randomized, double-blind cross-over design was used to determine the reliability of Meter 2 (Keto-Mojo, 952 School Street 212, Napa, CA 94559) and agreement and diagnostic performance compared to the validated Meter 1 (Precision Xtra, Abbott Laboratories, 100 Abbott Park Road, Abbott Park, IL 60064) in the assessment of blood ketone and glucose levels. To test varied blood ketone and glucose levels, participants visited the laboratory three times (separated by 1 week) and randomly consumed one of three similarly flavored supplements mixed in 400 mL of water: maltodextrin with no ketones which acted as the placebo, racemic ketone salts (D,L-beta-hydroxybutyrate), and natural ketone salts (D-beta-hydroxybutyrate). Each of the two ketone supplements contained 7 g of BHB. All supplements were 41 cal doses and coded as ‘A’, ‘B’, or ‘C’, and the investigators and participants were all blinded to the supplement consumed at each trial until completion of the study*.*

Participants visited the laboratory for a familiarization visit and then a total of three times for data collection with a one-week washout period between visits. The familiarization visit included written informed consent and a health history questionnaire. This study was approved by Augusta University’s Institutional Review Board and all procedures performed were in compliance with institutional guidelines.

### Participants

An estimated required sample size was calculated based on recommendations provided by Bujang and Baharum [25]. Based on a minimum level of agreement of an ICC = 0.90, α = .05, power of 0.80, and 12 observations per subject (2 devices × 2 time points × 3 conditions), a sample size of 9–10 subjects was justified, with some extra subjects recruited to account for subject dropout. Thirteen overtly healthy participants (6 males, 7 females; 21.6 ± 3.0 years old) were recruited primarily from the University and all completed the study. Exclusionary criteria included 1) taking medications that affect blood pressure, insulin or renal function, 2) metabolic syndrome factors such as Type II diabetes, 3) pregnant, and/or 4) pre-existing health condition as indicated on a health history questionnaire.

### Protocol

Two Meter 1 s and packaged glucose and ketone test strips were ordered from a local medical supplier. Two Meter 2 s and glucose and ketone test strips were sent directly from the manufacturer. The meters and test strips were stored in a temperature-controlled laboratory, in a cabinet away from water sources. All measures were taken in the same laboratory at 136 ft in elevation. Measures were taken on a clean work station where food and/or drinks were not allowed.

During the familiarization visit, participants signed the informed consent document, filled out a health history questionnaire, took a pregnancy test if female, and were provided a nutritional bar (Clif Bar, Clif Bar & Company, Emeryville, CA 94608; 250 kcal, 4.5 g fat, 45 g total carbohydrates, 4 g fiber, and 9 g protein) to eat for dinner the night before the first data collection visit. Participants were instructed to fast for 10 h, and withhold from exercise, caffeine, and nicotine for 12 h prior to the subsequent data collection visit. Verbal confirmation of participant understanding was obtained when the instructions were provided, and participants were again reminded of the nutrition and pre-testing instructions the day before visits to the laboratory. Adherence to these instructions was confirmed again on the day of testing before data collection proceeded.

The three data collection visits followed the same protocol and were separated by a one-week washout period. Upon entering the laboratory, females were given a pregnancy test. Baseline blood ketone and glucose levels were measured via a finger prick. Height and weight were recorded. Participants then consumed either the placebo, racemic ketone salts, or natural ketone salts (coded ‘A’, ‘B’, or ‘C’) according to a previously determined randomized and counterbalanced order. Drinks were mixed and consumed away from the testing site to ensure contamination with the meters did not occur. Blood ketone and glucose levels were measured 30 min and 60 min after the supplement was consumed, respectively. A nutrition bar was provided again at the first two data collection visits to be consumed for dinner prior to the subsequent visit.

### Blood meter measures

Prior to sampling blood, participant hands were washed thoroughly to reduce contamination and clean gloves were worn by investigators. The finger used for sampling was cleaned in a circular motion with an alcohol wipe and left to air dry for 30 s. Once pricked with a lancet, the first drop of blood was wiped away and the following drops of blood were immediately collected. During collection, the finger was only pressed with enough pressure to obtain a sufficient amount of blood for an adequate reading. The finger was not pressed again until the subsequent strip was ready for blood collection. This allowed less time for air to touch the exposed blood. At baseline, ketone and glucose levels were measured with both Meter 1 and Meter 2 twice in an alternating fashion. After consuming the supplement, ketone levels were measured again at 30 min and glucose at 60 min with both Meter 1 and Meter 2 twice in an alternating fashion. Multiple investigators measured glucose and ketone levels to allow less than 2 seconds between blood measures at each time point.

### Statistical analysis

All statistical analyses were conducted using SPSS version 25 (SPSS Inc., Chicago, IL). The test-retest reliability of both meters was evaluated to ensure consistent readings within the same meter in the population and conditions used in this study. Test-retest reliability of Meter 1 and Meter 2 for measuring ketone and glucose levels were calculated using intraclass correlation coefficient (ICC) estimates and their 95% confidence intervals based on a mean rating (k = 2), absolute agreement, 2-way mixed-effects model.

Interrater reliability between the two meters was similarly analyzed for ketone and glucose readings by calculating ICC estimates and their 95% confidence intervals using the average of the two readings for each meter at each time point. Thus, a single-rating, absolute agreement, 2-way mixed-effects model was used. All results are presented in the text as (estimated Average Measures ICC; 95% confidence interval) and are interpreted according to the guidelines suggested by Koo and Li [26]. Additionally, Bland-Altman plots were constructed for ketone and glucose measurements taken using Meter 1 and Meter 2. The average of the two readings taken at each time point were used to calculate the bias (average difference between meter measurements) and upper and lower limits of agreement (bias ±2SD of bias) between meters for measuring ketones and glucose across the range of observed values. The bias and limits of agreement were then used to construct the Bland-Altman plots to visually assess agreement between Meters 1 and 2, since correlation coefficients are not an acceptable indication of agreement [27].

For ketone measurement only, diagnostic performance of Meter 2 vs. Meter 1 for identifying NK was assessed via ROC curve construction and AUC analysis. Sensitivity and specificity test characteristics were also computed with the crosstabs function using a threshold BHB value of 0.5 mM. Sensitivity was calculated as the proportion of samples measured by Meter 1 with BHB concentration ≥ 0.5 mM that were similarly identified by Meter 2. Specificity was calculated as the proportion of samples measured by Meter 1 with BHB concentration < 0.5 mM correctly diagnosed as such by Meter 2 [28]. The level of 0.5 mM was chosen because it is typically used as a minimum blood ketone concentration for indicating NK [2, 17].

The meters in this study measured glucose in a healthy sample of adults and were not evaluated for use in a clinical population. Therefore, an error grid analysis for glucose measurement was not constructed to evaluate the clinical significance of the accuracy of Meter 2.

## Results

Time points for which there was only one data point (due to device malfunction or user error) were not included in the test-retest analyses. These single data points were used for the interrater reliability analyses as a representative average of the two points at that time.

### Meter 1 and meter 2 reliability

Complete test-retest reliability statistics including ICC estimates, *p-*values, and 95% confidence intervals are listed in Table 1. Test-retest reliability was considered excellent for measuring ketones with both Meter 2 and Meter 1. Test-retest reliability was considered good for measuring glucose with Meter 2 and excellent for Meter 1. The test-retest reliability ICC values were significantly different from 0 (*p* < .0005) in all cases.

**Table 1:** Reliability estimates for measuring ketones and glucose

	Ketones			Glucose		
	ICC	*p*	CI_95_	ICC	*p*	CI_95_
Test-Retest Reliability						
Meter 1	.993	< .0005	.989–.996	.920	< .0005	.872–.950
Meter 2	.974	< .0005	.959–.984	.873	< .0005	.788–.924
Interrater Reliability	.968	< .0005	.942–.981	.809	< .0005	.642–.893

### Meter 1 and meter 2 agreement

**Fig. 1:**
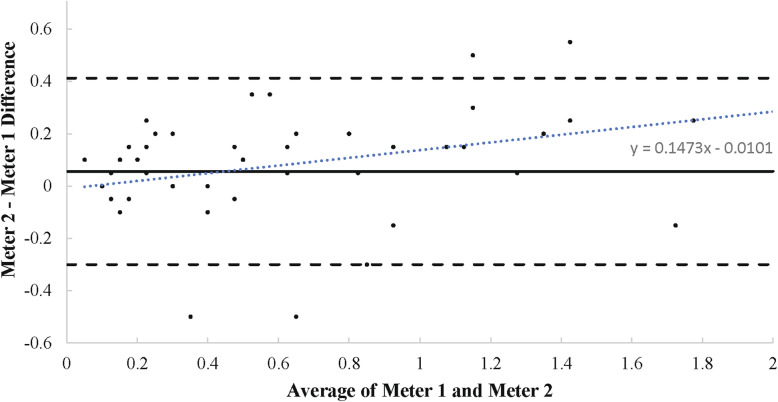
Meter 1 and Meter 2 Agreement – Ketones. Bland-Altman plot showing agreement between two meters for ketone measurement. Each data point is plotted on the graph with the x-value as the average ketone reading of the two meters and the y-value as the difference between the two meters for the respective reading. A y-value closer to 0 indicates a higher level of agreement. The level of agreement is assessed throughout the range of average ketone levels analyzed. The solid line represents the bias, or average difference in measurement between meters. The two dashed lines represent the limits of agreement (bias ±2SD of bias). The dotted line is the line of best fit for all of the graphed data points, described by the linear equation

Complete interrater reliability statistics including ICC estimates, *p-*values, and 95% confidence intervals are listed in Table 1. Interrater reliability between Meter 1 and Meter 2 was excellent for measuring ketones. As indicated in the Bland-Altman plot (see Fig. 1), bias between measurements was 0.056 mM with upper and lower limits of agreement equal to 0.412 and − 0.300 mM. Meter 2 was higher than Meter 1 by 0.056 mM on average, and the difference between meters ranged from − 0.300 to 0.412 mM for 95% of readings.

**Fig. 2:**
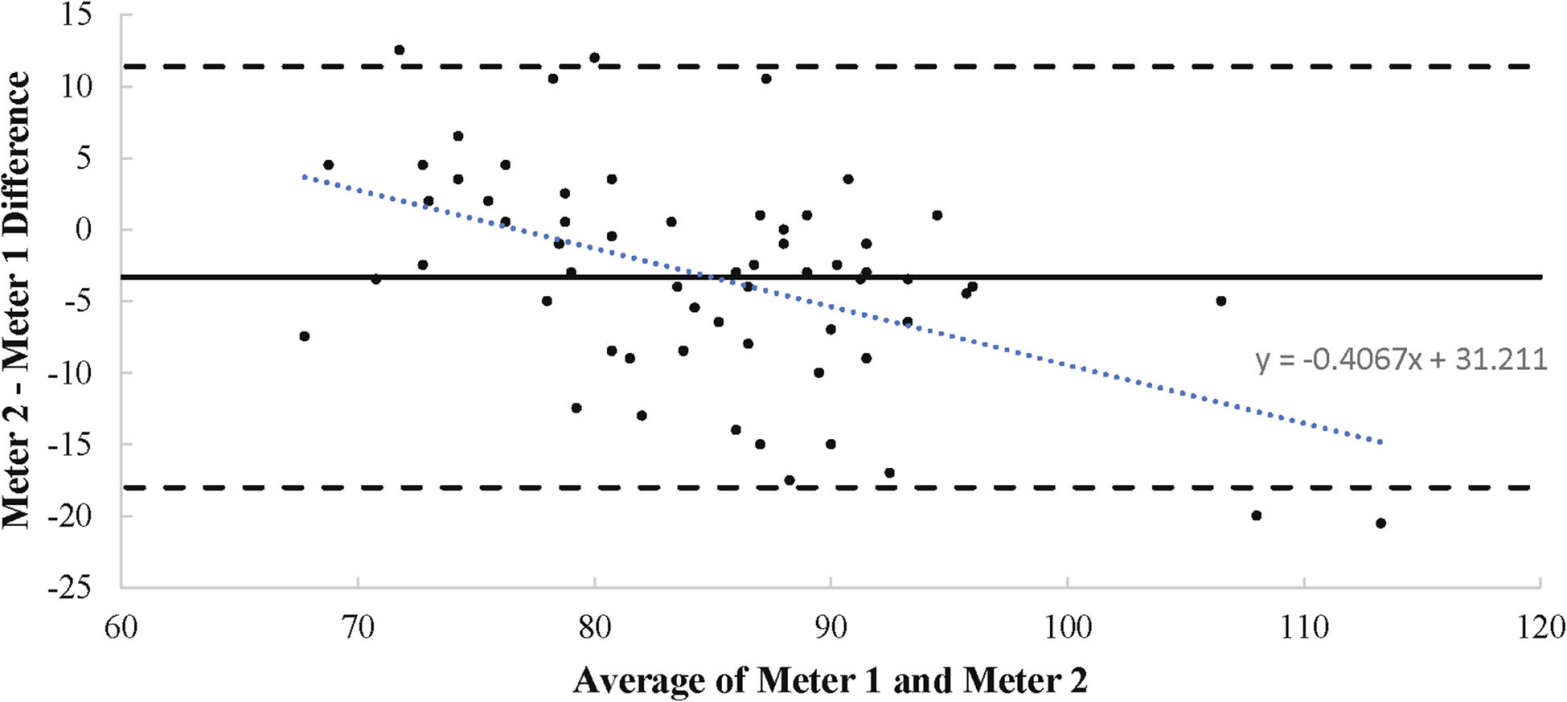
Meter 1 and Meter 2 Agreement – Glucose. Bland-Altman plot showing agreement between two meters for glucose measurement. Bland-Altman plot showing agreement between two meters for glucose measurement. Each data point is plotted on the graph with the x-value as the average glucose reading of the two meters and the y-value as the difference between the two meters for the respective reading. A y-value closer to 0 indicates a higher level of agreement. The level of agreement is assessed throughout the range of average ketone levels analyzed. The solid line represents the bias, or average difference in measurement between meters. The two dashed lines represent the limits of agreement (bias ±2SD of bias). The dotted line is the line of best fit for all of the graphed data points, described by the linear equation

Interrater reliability between Meter 1 and Meter 2 was considered good for measuring glucose (ICC = .809; .642–.893) and was significantly different from 0 (*p* < .001). Bias between measurements was − 3.322 mg/dL with upper and lower limits of agreement equal to 11.367 and − 18.012 mg/dL. Meter 2 was lower than Meter 1 by 3.322 mg/dL on average, and the difference between meters ranged from − 18.011 to 11.367 mg/dL for 95% of readings (see Fig. 2).

### Sensitivity, specificity, and AUC analysis for detecting NK

Sensitivity and specificity of Meter 2 to detecting NK (BHB ≥ 0.5 mM) as identified by Meter 1 was 90.5 and 92.2%, respectively. Complete sensitivity and specificity statistics can be found in Table 2. Area under the ROC curve analysis was performed to evaluate the ability of Meter 2 to discriminate between measurements above and below the 0.5 mM threshold of NK identified by Meter 1. The area under the ROC curve was .913 (95% CI, .828–.998) which is considered an excellent level of discrimination [28].

**Table 2:** Sensitivity and Specificity for Diagnosing Nutritional Ketosis

		Meter 1		Total
		No NK	Yes NK	
Meter 2	No NK	**47**	2	49
		**92.2%**	9.5%	68.1%
	Yes NK	4	**19**	23
		7.8%	**90.5%**	31.9%
Total		51	21	72
		100%	100%	100%

Sensitivity and specificity values (the proportion of tested cases in which Meters 1 and 2 agreed on the outcome of NK) are in bold.

## Discussion

The main objective of this study was to evaluate the agreement between two commercial ketone and glucose testing devices in healthy human subjects: Meter 1 (Precision Xtra, Abbott Labs) that has been validated in human and animal subjects, and Meter 2 (Keto-Mojo) which is more affordable but has not been validated. A second objective of this study was to assess the diagnostic performance of Meter 2 vs. Meter 1 for identifying NK at a level of BHB ≥ 0.5 mM.

### Ketones

Both Meter 1 and Meter 2 displayed excellent test-retest reliability for BHB measurement, and interrater reliability between the two meters was also considered excellent. The bias, or average difference between readings for Meter 1 and Meter 2, was 0.056 ± 0.18 mM. That is, Meter 2 produced ketone measurements that were 0.056 mM higher than Meter 1, on average. The variability of each reading was large relative to the bias, but differences between Meter 1 and Meter 2 appeared consistent at all levels of measurement according to the Bland-Altman plot.

In addition to reliability, the practical significance of the level of agreement between devices must be considered in the context of the population of interest. A meter designed for persons with uncontrolled Type 1 diabetes, for example, would need to detect pathological ketoacidosis in which blood pH drops to dangerously low levels and can be lethal [23]. Higher BHB readings reflect a greater potential for harm, so precise and accurate measurement is paramount over a wide range, especially those greater than 10.0 mM [23].

Detecting NK, which is encountered at a ketone level from 0.5–3.0 mM, does not bear the same stringent standard of measurement [29]. Ketoacidosis typically does not result from NK, even after prolonged starvation [30]. In addition, there is little evidence showing a dose-response relationship between BHB levels in the blood and metabolic or performance adaptations to the ketogenic diet. Practically speaking, a ketone measurement in healthy individuals that carries a small-moderate amount of error would not have lethal or performance-based repercussions.

Practical use of a ketone meter for athletes and those adhering to a ketogenic diet would be as simple as confirming that one is (or is not) in a state of NK. Confirming NK (BHB ≥ 0.5 mM) is an indication that the diet and exercise routine are sufficient to induce ketogenic adaptations, while a measurement to the contrary (BHB < 0.5) would indicate that a change in diet is appropriate to expedite ketosis (e.g. further limiting carbohydrate intake). In this sense, a useful measurement device should correctly discriminate nutritionally induced ketogenic state by displaying high sensitivity (to correctly detect when one *is* in a state of NK) and high specificity (to correctly identify persons *not* in a state of NK).

Sensitivity and specificity characteristics indicate that Meters 1 and 2 reached the same practical decision (NK or no NK) in roughly 90% of the cases. The sensitivity would be expected to increase further following complete adaptation to the ketogenic diet as resting BHB levels increase. BHB levels of up to 3.0 mM are common following adaptation to the KD [17, 31]. The greatest amount of error generated by Meter 2 (− 0.300 mM, the lower limit of agreement used in the Bland-Altman plot) would still result in a confirmatory NK value (≥ 0.5 mM). This would be the case even taking into account the bias between Meter 1 and the gold standard of measurement, which is reported to be 0.5 mM [19]. BHB levels reasonably above the lower limit for NK of 0.5 mM (i.e. close to 3.0 mM) would not be interpreted differently by Meters 1 and 2 from a practical standpoint. That is, the bias observed between devices in this study (0.056 ± 0.18) would most likely not have practical consequences.

False positive results could be encountered as well, in which an athlete is not in NK but the meter indicates that they are. Given that resting ketones in a fasted state are reported to reach 0.1 mM [6, 32], and a reading 0.4 mM higher than that (the upper limit of agreement between Meters 1 and 2) could be interpreted as being a state of NK. Early in the KD, before full adaptation has taken place, it may be more likely for Meter 2 to make a false positive (incorrectly indicating NK) or a false negative (incorrectly indicating an absence of ketogenic adaptation). For this reason, it may be best to take multiple measurements at a given time point early on in the adaptation phase (i.e. within the first week of diet adherence) to verify ketone levels and avoid false readings that could impact the decision made regarding a diet. Following chronic adaptation to the KD (> 2 weeks), the resulting higher BHB levels would make Meter 2 less vulnerable to errors when seeking to confirm NK. Importantly, taking multiple measurements with Meter 2 would still be substantially more cost-effective than a single reading with Meter 1.

### Glucose

Glucose levels in the blood decrease during adaptation to the KD as glycogen is depleted and KBs are increased. However, blood glucose levels typically do not fall to dangerously low levels in part due to gluconeogenesis from amino acids and glycerol [33], as well as glucose sparing in favor of KBs [34]. Athletes concerned about hypoglycemia and possible lightheadedness and fatigue may opt to measure circulating glucose levels in the early stages of the KD for this reason.

Although interrater reliability between the two devices was considered good [26] and bias was relatively small (− 3.322 ± 7.34 mg/dL), visual inspection of the differences on the Bland-Altman plot shows dramatic differences between Meters 1 and 2 depending on the level of measurement. Specifically, Meter 2 tended to produce higher readings than Meter 1 at values < 80 mg/dL and lower readings than Meter 1 at values > 80 mg/dL.

Meter 2 had the highest apparent agreement with Meter 1 at glucose values near 80 mg/dL, which is close to the commonly reported upper bound for resting fasted glucose levels in KD adapted individuals [6]. A reading using Meter 2 taken from someone in the early stages of NK would likely be lower than this value, and therefore susceptible to more error, producing a higher observed reading than the true value. In other words, hypoglycemia is less likely to be detected with Meter 2 when present than with Meter 1. This discrepancy between devices could be due to the fact that Meter 1 and Meter 2 were designed for use in different populations (clinical vs. athletic).

Meter 2 is not recommended for precise glucose measurement in athletes adopting the KD due to the large variation from Meter 1 at glucose levels likely to be encountered by athletes in NK. It may be more appropriately used to measure glucose following the initial adaptation period when glucose levels return to near normal levels.

### Limitations

The findings of this study are limited by the fact that a field measurement device was used as the reference method for evaluating reliability, rather than the gold standard laboratory method. It is therefore more difficult to accurately interpret the measurements made by Meter 2, despite the fact that Meter 1 displays acceptable agreement with laboratory measurement of ketones up to 3.0 mM [19]. Nonetheless, the measurement of ketone levels using Meter 2 may be deemed acceptable for the purposes of indicating the presence or absence of NK, which requires less precision but is still meaningful.

Another limitation of these findings is that ketone readings above those normally encountered in NK (> 3.0 mM) were not measured or evaluated. It would be interesting to observe the accuracy of Meter 2 at these higher levels. There is little to no evidence reporting that different BHB levels are indicative (or causal) of a greater magnitude of adaptations. If any level of BHB indicative of NK (≥ 0.5 mM) results in the same level and speed of adaptation to the KD, then agreeability of the meters above NK levels are not crucial in determining adaptation. Future studies should investigate if different levels of KBs indicate different levels of adaptation to the KD.

All test strips used for the respective meters were from the same lot, so we cannot comment on the reliability of each device using strips from different lots. It is important to keep in mind that the environment that test strips are transported and stored in can impact the integrity of the strips and hence their reliability and validity. For example, if strips are stored in hot (> 39 C) or frozen (<− 20 C) environments that are not uncommon during sea or parcel freight, they may have a diminished ability to make accurate ketone or glucose readings. For this reason, strips from different lots may yield inconsistent results. It is recommended that test strips are purchased directly from the manufacturer and that the appropriate measures are taken to ensure a well-controlled shipping environment and resulting reliability of the test strip.

## Conclusion

For the purpose of identifying nutritional ketosis, the Keto-Mojo device (Meter 2) is a reliable and accurate portable blood ketone measurement device that is more cost-effective than another commonly used portable ketone meter, the Precision Xtra (Meter 1). The acceptable measurement error, consistent level of agreement, and high diagnostic performance of Meter 2 compared to Meter 1 makes it appropriate for use by recreational or professional athletes interested in verifying that they are in a state of NK on a daily basis.

## Data Availability

The data sets used and/or analyzed during the current study are available in the Open Science Framework repository, https://osf.io/5eu9f/

## Abbreviations

ICC: Intraclass correlation coefficient
BHB: Beta-hydroxybutyrate
NK: Nutritional ketosis
FFA: Free fatty acid
